# Call of the Maryland Academy of Science and Literature

**Published:** 1835

**Authors:** 


### Art. III.—Call of the Maryland Academy of Science and Literature.

We have lately received a Circular from this Association, designed to make known the destruction of its collections by fire. Through its President, P. Macaulay, M. D., and its Secretary, J. Mason Campbell, the Academy makes an appeal to similar associations, and to men of science throughout the Union, which we trust will not be in vain. All institutions for the cultivation of our natural history, should be regarded as members of one brotherhood, and stand ready to assist each other, in calamities which cannot be repaired, like those of ordinary property, by the expenditure of money. But we shall let the Academy speak for itself, in the following extract:

"The Maryland Academy of Science and Literature, having lately had the misfortune to lose its valuable Museum and Library by fire, respectfully and earnestly appeals to those who feel a common interest in its pursuits, for aid in repairing its loss.

The assiduous labor of nearly thirteen years had amassed for it a large number of costly literary and scientific works, an extensive collection of minerals, specimens in almost every department of Natural History, and many coins, medals and antiquities. In commencing anew the work of accumulation, much, it is persuaded, can be done by the contributions of the public and similar Institutions. Most of such societies and many individuals possess superfluous specimens and duplicates of Books, of but little or limited use to themselves, while of value to those who are without and need them. To associations or individuals who have it thus in their power to aid the Academy, it cannot be necessary, for securing that aid, to address any considerations. The cause of science and literature, is one of no merely local or private concern, but possesses an interest and importance of the widest character, and its followers, in what place soever they urge its extension, must be counted as of one brotherhood. To the same end all co-operate, and it no doubt must be the impulse of feeling, as it certainly is the dictate of duty, that each, where it is possible, shall lend encouragement and assistance to the other.

The collections which the Academy seeks to make, embrace all that can claim the attention of the literary and scientific. They include minerals, shells, fossils, specimens in Natural Science, books, coins, aboriginal antiquities, maps and documents illustrative of the history, geography, or literature of any portion of the world, and in particular of Maryland. Unpublished barometrical or thermometrical observations—descriptions of celestial or terrestrial phenomena, and State Statistics which have never been given to the world are likewise among the means of information which it seeks to gather and make useful. Donations coming under the above description, or aught else that can attract the observation of the curious, or stimulate the inquiries of the learned, will be thankfully received and acknowledged."

